# A Novel EMG-Based Hand Gesture Recognition Framework Based on Multivariate Variational Mode Decomposition

**DOI:** 10.3390/s21217002

**Published:** 2021-10-22

**Authors:** Kun Yang, Manjin Xu, Xiaotong Yang, Runhuai Yang, Yueming Chen

**Affiliations:** School of Biomedical Engineering, Anhui Medical University, Hefei 230032, China; yk15656066591@163.com (K.Y.); xumanjin@ahmu.edu.cn (M.X.); yxt6686637@163.com (X.Y.); yangrunhuai@ahmu.edu.cn (R.Y.)

**Keywords:** sEMG, MVMD, separable convolution neural network, hand gesture recognition, two-stage framework

## Abstract

Surface electromyography (sEMG) is a kind of biological signal that records muscle activity noninvasively, which is of great significance in advanced human-computer interaction, prosthetic control, clinical therapy, and biomechanics. However, the number of hand gestures that can be recognized is limited and the recognition accuracy needs to be further improved. These factors lead to the fact that sEMG products are not widely used in practice. The main contributions of this paper are as follows. Firstly, considering the increasing number of gestures to be recognized and the complexity of gestures, an extensible two-stage machine learning lightweight framework was innovatively proposed for multi-gesture task recognition. Secondly, the multivariate variational mode decomposition (MVMD) is applied to extract the spatial–temporal features from the multiple channels to the EMG signals, and the separable convolutional neural network is used for modelling. In this work, the experimental results for 52 hand gestures recognition task show that the average accuracy on each stage is about 90%. The potential movement information is mainly contained in the low-frequency oscillator of the sEMG signal, and the model performs better with the low-frequency oscillation from the MVMD algorithm on the second stage classification than that of other decomposition methods.

## 1. Introduction

A human–machine interface (HMI) is defined as a feature or component of a certain device or software application that enables humans to engage and interact with machines [1]. In short, it refers to use software or algorithms to entrust the ability of understanding intentions from human to machines. Human hands are capable of skilful and intricate movements. Due to the multiple degrees of freedom, hands give humans the capacity to manipulate objects and even replace verbal communication through gestures [2]. Hand gestures offer the way to interact, control with devices and machines naturally. With the development of the sensor technology, non-contact sensor as computer-vision [3], radar [4] and Wifi [5] and contacted device as Myo armband [6] (Thalmic startup) and inertial measurement unit (IMU) provide various solutions for hand gesture recognition [7].

As image processing technology has developed, vision-based hand gesture recognition has become a low-cost and easy method to implement, but it is extremely susceptible to background noise such as colour overlapping, lighting and camera angle, making it difficult for a machine vision-based recognition method to obtain ideal recognition accuracy [8]. Gesture recognition based on wearable sensors are able to better adapt to the scene. In recent years, many researchers have explored in depth the sensor-based wearable framework, IMU, with three-axis acceleration, gyroscope, magnetometer mounted on the arms, legs and fingers for data acquisition [9,10] and data pre-processing for denoise and normalization for machine learning. Feature extraction and classification has also been performed to build a mathematical model for recognizing hand gestures. However, this method is costly and not friendly to the disabled. A large number of wearable sensors are also inconvenient for users and interfere with the daily life.

sEMG is a kind of biological signal that records muscle activity. HCI based on biopotential is a new type of human–computer interaction technology that utilizes electrical signals from the body to directly establish channels between external physical devices and humans [11]. sEMG is often used to record and analyse the bioelectrical signals generated by muscle activities and the sum of action potentials of multiple motor units that contain potential body movement information [12]. sEMG is safe, simple and non-invasive, as it captures and transmits with electrode arrays [13].

sEMG is a nonlinear, non-stationary, low-frequency signal and the amplitude is random in nature, but it is easily disturbed by ECG signals, cardiac artifacts, and external noise during acquisition. These factors make it difficult for analysis [14]. Previous research has explored the time-domain feature [15], which consists of IAV (integrated absolute value), RMS (root mean square), WL (wavelength) and it has been proven that time domain features are closely related to hand gestures. The frequency domain features contain spectrum median, energy spectrum, and power spectrum features [16]. Although with time-domain and frequency-domain techniques we can usually extract low-level features from a fixed window, these features do not represent the true non-stationarity of real-world signals and capture only the global information. STFT (short time fourier transform) extract the time–frequency domain feature from an sEMG signal with fixed window size, but the window width is difficult to set and greatly effects the analysis result. The discrete wavelet transform (DWT) [16,17,18] decomposes the signal into a set of functions which are orthogonal to its translation and scaling. The difficulty for the wavelet method is the parameters including the mother wavelet, the scale, and the level. These are empirically determined to match with the properties of the input signal. Another time-frequency analysis method is fully data-driven with fewer settings or specificities. Empirical mode decomposition (EMD) is proposed to analyse biomedical signals and limited intrinsic mode functions (IMFs) shows the dynamic properties of the signal. EMD-based methods have shown equal performance with simple parameters to DWT algorithm. By adding white noise to the original signal, ensemble empirical mode decomposition (EEMD) is proposed to deal with mode-mixing problem. Both EMD and EEMD suffer the accumulation of estimation error and limited frequency resolution. Variational mode decomposition (VMD) [19] is an adaptive and non-recursive method; each mode is around its centre frequency with limited band and less spectral overlapping. As to analyse multiple channel signals, multivariate empirical mode decomposition (MEMD) and multivariate variational mode decomposition (MVMD) algorithms are proposed to handle multiple channel signals such as ECG, EEG [20], sEMG [2].

The feature vector formed by the above method is often used to establish gesture recognition models. For the classification model, there are conventional machine learning methods such as support vector machine (SVM) [17,21], random forest (RF), native bayes (NB) and clustering methods. These are lightweight and largely depend on manually extracted features. Manually extracted features are limited by human knowledge and experience, resulting in limited accuracy. It is necessary to extract a large number of features to achieve satisfactory recognition accuracy, this process therefore takes a lot of time and effort to find feature vectors to achieve ideal accuracy. The number of hand gestures to be recognized is also limited by this method. To deal with the above problems, researchers have proposed end-to-end neural networks to handle sEMG; they simply regard sEMG signal as 2-D images [22] and take the image as the input of the neural network. The end-to-end neural network can learn features and classify automatically, avoiding the deficiencies of human knowledge and eliminating the hassle of finding suitable features vectors. Theoretically, increasing the number and layers of neurons is beneficial to the final result, but the performance improvement is actually limited. As the number of hand gestures rises, the performance of the neural network reduces significantly. The additional neurons also lead to greatly invalid calculation, therefore it is insufficient to deal with sEMG images simply by the image processing method. The high-power consumption makes it impossible to transplant this model onto an embedded device.

In order to address these issues, we proposed a two-stage multiple hand gesture recognition framework. First, all the hand gestures were divided into several superclasses (basic movements of fingers, isometric, isotonic hand configurations, basic wrist grasping, and functional movements) with similar mechanical properties. This process was easy to achieve by conventional time–domain features. Second, we trained models for each superclass, after applying MVMD algorithm to sEMG signal, as the low-frequency oscillation of sEMG is regarded as the input of neural network. Then, we trained the input signal with separable convolutional neural network (CNN). This process is lightweight and equal to the performance of CNN. Due to the obvious mechanical characteristics of hand gestures, it is easy to obtain the superclass of the hand gesture just with the time–domain features vector. MVMD algorithm extracts the temporal and spatial characteristics of multiple channel signals, which not only preserves the correlation between the channels, but also highlights the time-frequency characteristics in each channel.

The rest of the paper is organized as follows. Section 2 reviews the related algorithm such as label calibration and MVMD algorithm. Section 3 describes the detailed data flow on each stage. Section 4 gives the dataset description and experiment method. Section 5 gives the experiment result and analysis. At the end of this paper in Section 6, we conclude our work and point out prospects for the future.

## 2. Related Algorithm

### 2.1. Relabel

Human response times and attention spans eventually trigger some misalignment, as carried out by the subject between the stimulus video and the actual movement. In order to minimize this label “noise”, the hard threshold method based on the Teager energy operator is used to locate active sEMG. The Teager energy operator tracks the modulation energy and captures the instantaneous amplitude, frequency of sEMG [23]. In this paper, the Teager energy operator highlights the active sEMG signal and weakens the rest, which can significantly distinguish the active state from the full record. The calculation methods of the Teager energy operator in the discrete domain and continuous domain are shown in (1) and (2).

(1) $$\varphi (x(t))=x^{\prime }{\left(t\right)}^{2}-x(t)x^{\prime \prime }(t)$$

(2) $$\varphi (x(n))=x{\left(n\right)}^{2}-x(n-1)x(n+1)$$

To identify the active segmentation as simply as possible, we calculated the Teager energy’s amplitude from sEMG. *E*(*t*) is expressed as (3), *E_average_* shows average value of Teager energy of all the channels. *N* is the setting width of the time window; the corresponding threshold is set to recognize the active samples point. If *E*(*t*) is greater than the set threshold and lasts for milliseconds, the active state begins. If *E*(*t*) is less than the set threshold and lasts for milliseconds, the active ends. This method is shown as function in (4).

(3) $$E\left(t\right)=\frac{1}{N}\sum_{i}^{i+N-1}E_{average}\left(i\right)$$

(4) $$label=\{\begin{array}{cc} active & E(t)>Thre\\ rest & E(t)<Thre \end{array}$$

### 2.2. Multivariate Variational Mode Decomposition

In the 1990s, Huang [19,24] put forward EMD as a pure data-drive algorithm. This method decomposes signals into several modes of unknown independent frequency bands, which is entirely different from the Fourier transformation and wavelet methods and is widely used today in signal processing, audio engineering, fault diagnosis, and biology. However, finding extreme points and stopping conditions are significant to the decomposed results, which reduces the algorithm’s robustness due to the lack of mathematical theory and freedom. In 2014, Konstantin proposed the VMD. Compared to the EMD algorithm, the VMD algorithm adaptively determines the relevant bands and estimates the “modes”, while balancing the errors between modes. For multi-channel data such as sEMG, EEG etc., MVMD analysis time-frequency characteristics decompose multi-channel signals into real “modes”. The main purpose of MVMD is to extract predefined K multivariate modulation oscillations from input multivariate signals $x\left(t\right)=[x_{1}(t),x_{2}(t),\ldots ,x_{C}(t)]$, as (5) [19].

(5) $$x(t)=\sum_{k=1}^{K}u_{k}(t)$$

where $u_{k}(t)=[u_{1}(t),u_{2}(t),\ldots ,u_{c}(t)]$. There are two strict constraints while finding the modulated oscillations ${\left\{u_{k}(t)\right\}}_{k=1}^{K}$. First, the sum of the bandwidth of each “mode” is as minimum as possible. Second, the sum of the modes recovers the original signal perfectly.

MVMD algorithm mainly consists of the following steps: (i) as for each mode, investigate the related analytic signal to obtain a unilateral frequency spectrum through the Hilbert transform method; (ii) move the frequency spectrum of each mode to the baseband by blending an exponential tuned to the respective approximate centre frequency. (iii) the bandwidth is eventually computed by the application of Gaussian smoothness to the demodulated signal. The above mentioned two constrained variational problems can be summarized as (6). $u_{+}^{k,c}$ is the vector analytic representation of $u_{}^{k,c}$ [25].

(6) $$\underset{\left\{u_{k,c}\},\{\omega_{k}\right\}}{\mathrm{minimize}}\left\{{\sum_{k}\sum_{c}\left\|\partial_{t}\left[u_{+}^{k,c}\left(t\right)e^{-j\omega_{k}t}\right]\right\|}_{2}^{2}\right\}\;\mathrm{subject}\;\mathrm{to}\sum_{k}u_{k,c}(t)=x_{c}(t),\;c\;=\;1,2,\ldots ,C.$$

There are multiple linear equality constraints in the bandwidth-constrained optimization problem, which can be solved by the augmented Lagrangian function. ADMM (Alter direction method of multipliers) approach converts a complex optimization problem into multiple straightforward sub-optimization problems. This process, the bandwidth minimization, is shown as follows: (i) initialize the first iteration of each mode in every channel, the centre frequency, and Lagrange multiplier; (ii) increment the corresponding iteration value to compute the next mode in the next channel; (iii) check whether the convergence is satisfied, otherwise increment the initialized parameters and repeat the above process until the convergence of the mode takes place [26]. The method of updating the mode, centre frequency and Lagrange multiplier is shown as (7)–(9). The final computed method of the convergence criteria is expressed as (10).

(7) $${\hat{u}}_{k,c}^{n+1}=\frac{{\hat{x}}_{c}(\omega )-\sum_{i<k}{\hat{u}}_{i,c}^{n+1}\left(\omega \right)-\sum_{i>k}{\hat{u}}_{i,c}^{n+1}\left(\omega \right)+\frac{{\hat{\lambda }}_{c}^{n}\left(\omega \right)}{2}}{1+2\alpha {\left(\omega -\omega_{k}^{n}\right)}^{2}}$$

(8) $$\omega_{k,c}^{n+1}=\frac{\sum_{c}\int_{0}^{\infty }\omega {|{\hat{u}}_{k,c}^{n+1}|}^{2}d\omega }{\sum_{c}\int_{0}^{\infty }{|{\hat{u}}_{k,c}^{n+1}|}^{2}d\omega }$$

(9) $${\hat{\lambda }}_{c}^{n+1}\left(\omega \right)={\hat{\lambda }}_{c}^{n}\left(\omega \right)+\tau \left({\hat{x}}_{c}\left(\omega \right)-\sum_{k}{\hat{u}}_{k,c}^{n+1}\left(\omega \right)\right)$$

(10) $$\sum_{k}\sum_{c}\frac{{\left\|{\hat{u}}_{k,c}^{n+1}-{\hat{u}}_{k,c}^{n}\right\|}_{2}^{2}}{{\left\|{\hat{u}}_{k,c}^{n}\right\|}_{2}^{2}}$$

## 3. Proposed Framework

### 3.1. The Data Flow in the Proposed Framework

**Figure 1:**
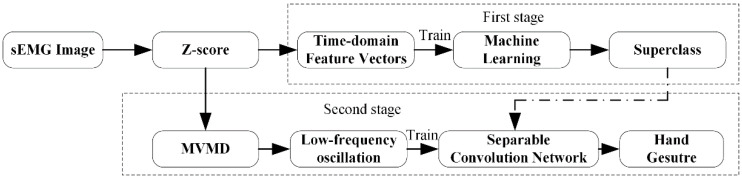
The data flow in the proposed framework. After pre-processing using the Z-score method. First, extract the time-domain features to find the superclass. Next, train the neural network with low-frequency oscillation to find the true label of the hand gesture.

Identifying a confusing item is not often achieved in one step, especially in the process of human cognition, which often involves long periods of thought and repeated confirmation [27]. Firstly, humans usually determine the approximate range of the items according to their obvious features. Next, we need to extract detailed features and think carefully before further understanding and judgement. Inspired by the human cognitive process [28], we designed two-stage gesture recognition model based on the sEMG when facing a multi-class gesture classification task. The proposed hand gesture recognition framework is shown in the Figure 1.

The core task is to train classifiers on each stage. On the first stage, we only need to know the hand gesture’s superclasses vaguely. Considering that different hand gestures have a certain force similarity in muscle activity, all hand gestures are simply divided into k superclasses, such as the basic movements of fingers, isometric, isotonic hand configurations, basic wrist grasping, and functional movements. Each superclass contains several different subclasses. This stage is easily completed using a classical machine learning method, such as SVM or RF.

The second stage is composed of k sub-classifiers model. To analyse hand gestures’ detailed features in each superclass, we use the MVMD algorithm to extract the spatiotemporal features from multi-channel sEMG signals. The extreme similarity of hand gestures leads to the complexity and difficulty for accurate recognition. It is necessary to take the classifiers based on neural networks such as CNN to achieve greater performance on the second stage. CNN shows strong performance in EMG gesture classification procedure [29,30,31]. A separable convolutional neural network model is proposed to replace CNN to avoid complexity and ensure the classifier’s performance. This is an extensible and lightweight gesture recognition framework consisting of specially designed classification models for different classification purposes on different recognition stages, not only ensuring the classification performance of the model but also significantly reducing the complexity of the framework.

### 3.2. The Classifier on the First Stage

**Figure 2:**
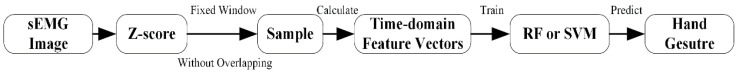
The detailed data flow on the first stage.

Previous off-line research has shown that the transient phase of sEMG signals contains the information of force [32,33]. For a wide variety of hand gestures, it is simple to divide them into several superclasses by the force level characteristics. Each superclass consists of several sub-classes with similar force level characteristics. Classical features such as MAV, RMS et al., have been closely related to the force level in the related work [34]. In general, grasping action’s force level characteristics are the most obvious, followed by wrist action, and finger action force level is the least. As Figure 2 shows, the sEMG signal was divided by fixed window without overlaps. We calculated the classic features shown in Table 1. After that the lightweight machine learning methods such as SVM, RF, DT, KNN, NB and Linear Discriminant Analysis (LDA) are tested on this stage for classification. The time window length of the sEMG image is another important factor while implementing the human–computer interface [35]. Therefore, different fixed window sizes are chosen to find the most suitable for hand gesture recognition. The optimal time window size obtained in this process is directly fed into the second-stage classification algorithm.

### 3.3. The Classifier on the Second Stage

Generally, the depth of layers and the number of neurons is essential to the neural network model’s performance. The parameters of extra network layers learn more internal rules of data sets, leading to overfitting and heavily computed burden. It is worth noting that the devices which ask for high real-time requirements cannot be satisfied. As a result, the previous proposed deep-learning hand gesture recognition frameworks are computationally intensive [29,36]. We put forward a two-stage framework based on neural networks to achieve high accuracy without computational costs.

Considering the difficulty of sub-classifier classification and the need to reduce the model’s complexity as much as possible. The layers and parameter of the proposed neural network is shown in Table 2. The model consists of three convolution layers, two pooling layers, and two dropout layers, dense layers and zero-padding layers. The dropout rate is 0.5. The detailed parameters setting in classical network and SCNN are shown as Para_1 and Para_2. Controlled by hardware computational level and the real-time gesture recognition requirements, we prefer to replace traditional convolution with separable convolution. This method can significantly reduce the computational complexity without performance loss. Separable convolution greatly simplifies the convolution process. When the kernels’ size is chosen (k × k), the filter is N, and the channel equals c, separable convolution can be simply divided into two steps [37]:

(i) The first step involves depth-wise convolution. Regarding the input data as composed of N channel data, we carried out single-channel convolution and then stacked them together again. This step resizes the data without changing the number of channels. The number of parameters trained is as (k × k × c).
(ii) The second step involves pointwise convolution. The characteristic image obtained by (i) is applied with the standard convolution twice. The convolution kernel size is 1 × 1, and the filter number of convolution kernels is the same as the channel number of the previous layer. The parameters trained can be calculated as 1*1*c*N. In comparison to the standard convolution, the parameter ratio of separable two-dimensional convolution can be reduced. The parameter ratio of separable two-dimensional convolution and standard convolution can be summarized as (11). In this experiment, the total parameters drop about 1/3, which is beneficial in a lightweight and real-time performance.
  (11) $$ratio=\frac{k\times k\times c\times N}{k\times k\times c+c\times N}$$

## 4. Dataset and Experiment Method

### 4.1. Dataset Description

**Figure 3:**
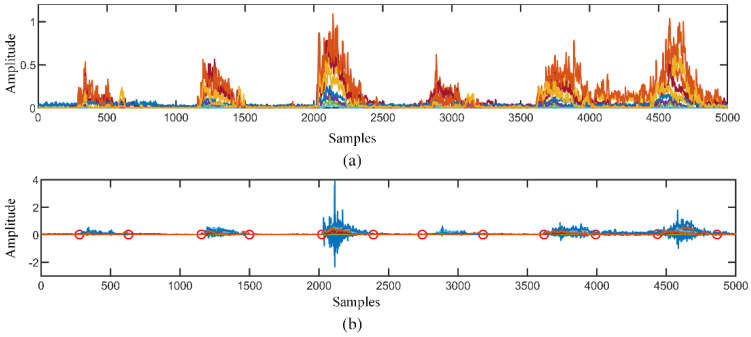
(**a**) shows the multiple channel sEMG signal with a different colour representing a different channel. The blue line in (**b**) is the average value of the Teager energy in all the channel, the red circles indicate the start and end time of the active signal.

The Ninapro data set [38,39] established by Idiap Research Institute has drawn the attention of researchers in the fields of machine learning, pattern recognition, clinical diagnosis, and neurocognitive science all over the world. They are devoted to the advanced science of robotic and prosthetic hands controlled by artificial intelligence. The Ninapro data set is now available on the website http://ninapro.hevs.ch (accessed on 1 August 2021). The Ninapro has already submitted eight sub data sets. DB-1 contains 52 different hand gestures covering basic movements of the fingers, isometric, isotonic hand configurations, and grasping and functional movements from 27 intact subjects. Figure 3 shows the representative three hand gestures in the superclass. During the data collection, the subjects have to repeat 52 movements 10 times, and the signal from the electrodes is acquired at a constant interval of 100 Hz. The signal of DB-1 is recorded with eight equally spaced around the forearm and two placed on the activity spots.

### 4.2. Experiment Method

The experiments of model training and prediction were carried out on a desktop computer equipped with i5-10400 CPU (16G DDR4 2666MHz, Intel Corporation, Santa Clara, CA, USA) and GTX 1050TI GPU (4G, Nvidia Corporation, Santa Clara, CA, USA). The desktop computer installed MATLAB 2010a (MathWorks, Natick, MA, USA), Python 3.8.3, sklearn 0.23.2, TensorFlow 2.3, and other software packages. MEMD, multivariate wavelet, and MVMD are implemented by MATLAB. Simultaneously, the classification algorithms are coded with the help of the Sklearn and TensorFlow-based machine learning framework.

Before the experiment setup, it was essential to normalize the data set by Z-score. After that, the fixed-size time window was used to segment the records without overlapping. After all the samples were randomly shuffled, the performance analysis of this system was conducted through a k-fold cross validation process. The whole data set is divided into k number of sub data sets, each subset contains an equal number of samples. The process was carried out by taking one subset for test set and then the remaining. The proposed work is validated by tenfold across the validation process. As to the second stage of classification based on neural network, the batch size was set to 256, the Adam optimizer was used for gradient descent, the initial number of iterations was set to 100. We used early stopping for training, and the other parameters were set by default. The models were optimized by adjusting the hyper parameters for better performance. The confusion matrix analysis was approximate for the classification results of each category. It expressed the correct classification of each category and the number and proportion of misclassification.

## 5. Result and Discussion

### 5.1. Relabel the Signal

As shown in Figure 3b, the red line shows the average value of the scaled multi-channel sEMG and the blue line represents the Teager energy of the average sEMG signal. compared with the original EMG signals, Teager energy highlights the action segmentation and smooths the rest segmentation. It is indicated that most of the labelled points are correctly located. As a result, the relabelled signal accurately represents the real motion state of subjects. This method can not only be used for the calibration data set in the online test, but also be regarded as an import judgment for a motion signal from the beginning.

### 5.2. Analysis sEMG by MVMD

**Figure 4:**
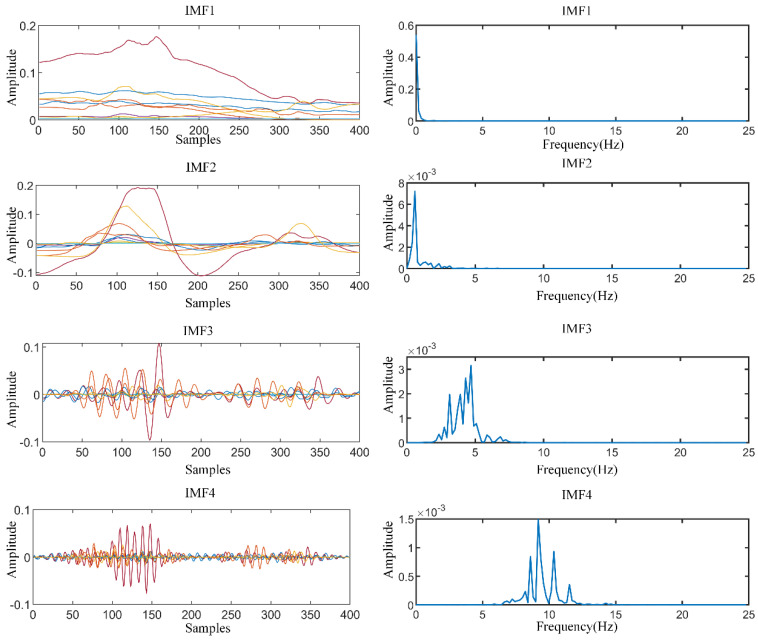
The decomposition modes and corresponding spectrum by MVMD.

We apply four-level decompositions by MVMD for sEMG and calculate the related frequency spectrum. The four oscillations of sEMG and the corresponding frequency spectrum is shown in Figure 4. The decompositions exhibit limited joint time and frequency resolution, which blurs the resulting T-F representation due to the Heisenberg uncertainty principle. The MVMD algorithm jointly transforms each channel into a series of stationary sub-signals and keeps temporal stationarity within-channel, as well as the spatial independence.

**Figure 5:**
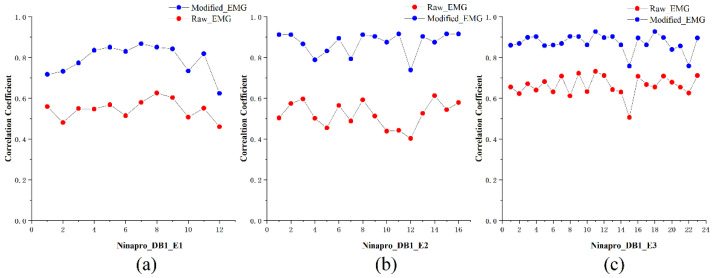
Average Pearson’s correlation coefficient of raw sEMG signal and the modified EMG signal for each hand gesture class in (**a**) Ninapro_DB1_E1, (**b**) Ninapro_DB1_E2, (**c**) Ninapro_DB1_E3. The coefficient value is higher for modified EMG signal than that of raw EMG signal signifying better signal quality and regularity throughout the Ninapro_DB1.

After obtaining multiple oscillations of multi-channel EMG signals, we compared the correlation between the low-frequency oscillation of each gesture action signal and the original signal and calculated the average value of the correlation coefficient for all of the subjects. For any hand gestures, the correlation of low-frequency oscillation is higher than that of the original signal. This is verified in all three sub datasets. As Figure 5 shows, the average coefficient value for the modified EMG is higher than that of the raw EMG in all the records. It is indicated that the modified EMG signal for each hand gesture obtained MVMD algorithm has higher correlation than the raw EMG signal, this result is calculated from 27 subjects from Ninapro_DB1.

### 5.3. Train the Model on the First Stage

**Figure 6:**
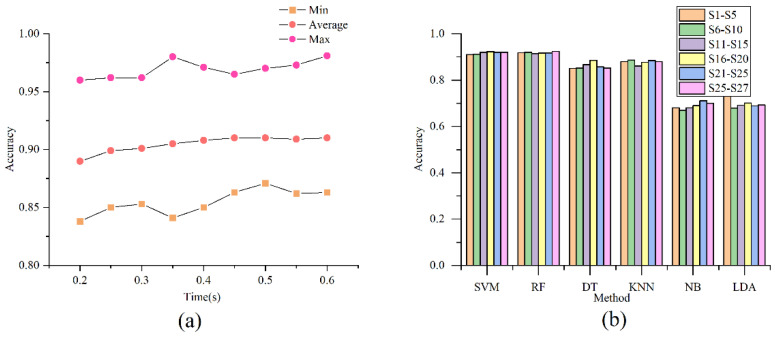
(**a**) shows the max, average and min value of the accuracy in different time windows, the accuracy rises with wider window in limited range. (**b**) indicates that train the feature vector with SVM, RF, DT, et al., the result varies, SVM and RF show better results than others.

Table 3 illustrates the average accuracy grouped by five subjects from the SVM method. By applying the classical machine learning method with force level features, we established the model mapping the classical features commonly utilized in the previous research to the three superclasses, including finger movement, wrist movement, and functional movement. It can be seen that the RMS and MAV are more relevant to the force level. The result of the model trained by the single feature reached 90.08% at highest, the selected features performed better and reached the highest average accuracy 93.05% on the group of the subject 5–10. The average accuracy on the first stage was more than 90%, Generally speaking, the potential force level in wrist movement was lower than that of functional movement, and greater than that of finger movement, As the experiment shows, recognizing the hand gesture vaguely by force level characteristic is feasible. Another import factor for hand gesture recognition is the length of the time window and stride window. The accuracy of different window length is shown in Figure 6a. When the window length is less than 400 ms, the accuracy rises, and then tends to be stable, the sample segmented by fixed window 400 ms ensures the performance of the accuracy and real time. As shown in Figure 6b, the classification performance of SVM and RF is relatively better. When the kernel function is set to “RBF”, the penalty item is between 15 and 20 [39], the gamma is “Scale”, the model has better performance than the default setting. We preferred to choose SVM for the first stage task as it only needed several support vectors for classification and less computation. The parameters storage and computation can be ignored compared to the neural network.

### 5.4. Train the Model on the Second Stage

**Figure 7:**
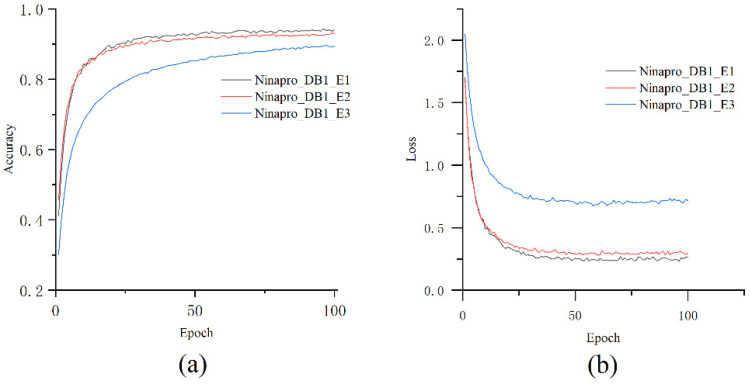
Training accuracy (**a**) and training loss (**b**) throughout the training procedure for Ninapro_DB1. In every case, the CNN learns the features of the proposed modified EMG signal and reaches about 90% in each sub data set.

**Figure 8:**
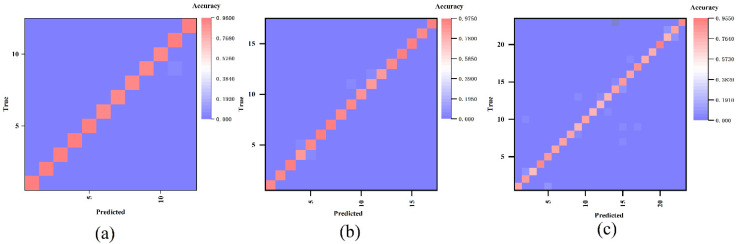
The confusion matrix analysis on the second stage (**a**–**c**); almost all the hand gestures in Ninapro_DB1 are classified correctly.

Unlike most gesture recognition frameworks, we ignored individual differences and mixed the samples of different subjects for training, which can fully reflect the wide applicability of our trained model. We used decompositions from the MVMD method as the neural network classifier’s input during the second stage classification training process. MVMD decompose the sEMG signal into independent frequency bands, and each represents different time-frequency characteristics. We conducted experiments on each sub data set in Ninapro_DB1, respectively. During the training process, the accuracy and the loss is as shown in Figure 7. the average accuracy of the final gesture recognition on NinaPro_DB1 is 93.95%, 92.9%, and 88.67%. As the three confusion matrixes in Figure 8 indicate, the majority of the hand gestures are correctly recognized in the three-sub data set, proving the high performance of the proposed recognition framework regardless the difference between the subjects. Although this model trained by dataset made up of all the subjects and ignored the difference caused by subjects, the results show equal performance to the individual training model existed.

**Figure 9:**
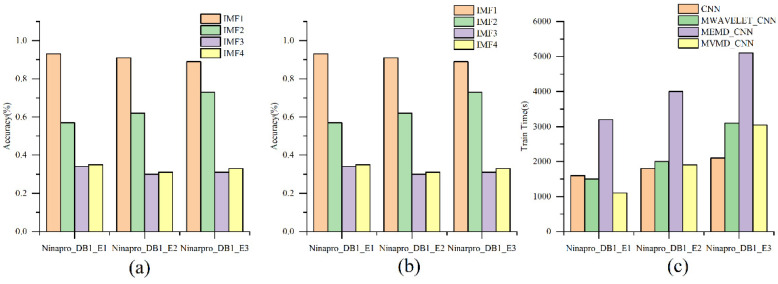
(**a**) shows the result trained by different IMF, the lower frequency oscillator contributes greatly (**b**,**c**) compared different decomposition methods in terms of accuracy and training time. MVMD shows a greater performance than others.

To further find the majority oscillators from all modes and reduce the burden of the hand gesture recognition model, we trained the model with every decomposed mode, respectively. The results are shown in Figure 9a. The lower frequency oscillator reached an average accuracy of 93.11%, 91.09%, 89.01%, which is same as that of the model trained by all the modes. The lower frequency oscillators contribute greatly to the result, which may be due to the fact that the amplitude and the other time-domain features is more relative to the hand gesture and training time. Finally, comparison methods are organized as follows:

(1) CNN: set the sEMG image as the input to train CNN.
(2) MWAVELET_CNN: decompose the sEMG image by wavelet 4-level into 3D-image, and then train the CNN model with the 3-D image.
(3) MEMD_CNN: decompose the sEMG image by MEMD into 3D-image, and then train the CNN model with the 3-D image.
(4) MVMD_CNN: decompose the sEMG image by MVMD into 3D-image, and then train the CNN model with the 3-D image.

As shown in Figure 9b, the decomposition algorithm achieves superior classification performance to the single CNN method; the accuracy is improved by 18.8%, 9.0% and 16.77% in E1.,17.6%, 9.0%, 18.7% in E2 and 16.3%, 10.3% and 13.6% in E3. For decomposition methods, the performance of MVMD and MEMD on different datasets is better than that of multivariate wavelet decomposition.

The common methods, such as wavelet transform, MEMD, and MVMD algorithm decompose into various modes which have a specific sparsity property. Different decomposition methods have their own advantages and disadvantages. As for sEMG, electrode position often indicates spatial characteristics. To compare the application of different decomposition methods in spatial feature extraction, we designed four groups of comparative experiments and evaluated decomposition methods’ performance.

Figure 9c shows the compared result of the training time of different decomposition methods. It can be seen that the time of MEMD is much longer than that of the other two methods. According to the observation data analysis. the number of “modes” obtained by different subjects vary from 15 to 20, while the MVMD and multivariate wavelet are only 4 and 5 as we set, respectively. Because of the number of modes, the training time of neural network becomes longer by MEMD_CNN.

## 6. Conclusions

A second-stage hand gesture recognition framework based on MVMD, inspired by the human cognitive process is put forward in this paper. This framework realizes high accurate recognition of multiple hand gestures and low computational cost compared with the existing hand gesture sEMG-based method. The introduction of MVMD improves the accuracy, and the second-stage perception module based on separable convolution significantly reduces the complexity of the model. As the experimental results have shown, it can be inferred that the proposed method of extracting the spatial-temporal characteristics of sEMG using MVMD is more precise than multivariate wavelet decomposition, and lowers the training time by half compared to MEMD. The two-stage model retains high accuracy for the multi-gesture recognition task without increasing the model’s complexity.

In the future, to further improve the real-time performance of the model in sEMG products, we will study in two directions. First, we will consider the feasibility of designing a proprietary FPGA chip to implement MVMD and a neural network model for hardware acceleration. Secondly, we will study the application of the “mode” decomposition method in biomedical signal processing, signal denoising, and feature analysis.

## Figures and Tables

**Table 1:** The selected classical features based on EMG for hand gesture recognition.

Index	DESC	Equation
RMS	Root mean square	$\sqrt{\frac{1}{L}\sum_{i=1}^{L}{\left(x_{i}\right)}^{2}}$
MAV	Mean absolute value	$\frac{1}{L}\sum_{i=1}^{L}\left|x_{i}\right|$
WL	Wavelength	$\sum_{i=2}^{L}\left|x_{i}-x_{i-1}\right|$
ZC	Zero crossing	$\sum_{i=1}^{L}f\left(x_{i}\right)=\{\begin{array}{cc} 1 & x_{i}*x_{i+1}<0\;\&\;\left|x_{i}-x_{i+1}\right|>T\\ 0 & \mathrm{otherwise} \end{array}$
SSC	Slope sign change	$\sum_{i=2}^{L-1}f\left(x_{i}\right)=\{\begin{array}{cc} 1 & \left\{(x_{i}>x_{i-1}\&x_{i}>x_{i+1})|(x_{i}<x_{i+1}\&x_{i}<x_{i+1})\right\}\\ & \&\{(\left|x_{i}-x_{i-1}\right|\geq T)\&(\left|x_{i}-x_{i+1}\right|\geq T)\}\\ 0 & \mathrm{otherwise} \end{array}$
DASDV	Difference absolute standard deviation value	$\sqrt{\frac{\sum_{i=1}^{L-1}{\left(x_{i+1}-x_{i}\right)}^{2}}{L-1}}$
WA	Willison amplitude	$\sum_{i=1}^{L-1}f\left(x_{i}\right)=\{\begin{array}{cc} 1 & \left|x_{i}-x_{i+1}\right|>T\\ 0 & \mathrm{otherwise} \end{array}$
VAR	Variance	$\frac{1}{L-1}\sum_{i=1}^{L}{\left(x_{i}\right)}^{2}$

**Table 2:** The parameters of the proposed neural network.

Layer	Size	Channel	Para_1	Para_2
Input	(30,10,1)	1	0	0
Convolution1	(3,3)	32	1184	73
Dropout	-	-	-	-
Convolution2	(3,3)	64	18,496	2400
Maxooling1	(2,2)	64	-	-
Convolution3	(3,3)	128	73,856	8896
Zeropadding	(0,1)	128	-	-
Maxpooling2	(2,2)	128	-	-
Flatten	-	-	-	-
Dropout	-	-	-	-
Dense	128	-	344,192	344,192
Softmax	12	-	1548	1548

**Table 3:** The recognition result grouped by five subjects on the first stage, the results are grouped and averaged.

Subjects	RMS	MAV	WL	ZC	SSC	DASDV	WA	VAR	RMS + MAV + DASDV
S1-S5	85.77	86.99	85.59	47.99	65.69	85.57	65.69	78.93	90.84
S6-S10	89.17	90.08	88.20	47.12	70.45	87.08	71.25	81.50	93.05
S11-S15	86.50	87.33	83.39	46.81	63.87	82.87	62.43	76.22	91.02
S16-S20	82.63	84.01	82.57	47.39	61.26	81.92	60.80	75.75	90.24
S21-S25	87.97	87.92	86.00	43.69	63.88	84.89	61.33	79.22	90.77
S26-S27	87.78	87.16	87.13	44.65	70.46	85.47	63.97	80.15	92.21

## References

[1] Cannan J., Hu H. (2011). Human-Machine Interaction (HMI): A Survey.

[2] Zhang Y., Chen Y., Yu H., Yang X., Lu W. (2020). Learning Effective Spatialoral Features for sEMG Armband-Based Gesture Recognition. IEEE Internet Things J..

[3] Kumar H., Honrao V., Patil S., Shetty P. (2013). Gesture Controlled Robot using Image Processing. Int. J. Adv. Res. Artif. Intell..

[4] Zhang Z., Tian Z., Zhou M. (2018). Latern: Dynamic Continuous Hand Gesture Recognition Using FMCW Radar Sensor. IEEE Sens. J..

[5] Abdelnasser H., Harras K.A., Youssef M. WiGest demo: A ubiquitous WiFi-based gesture recognition system. Proceedings of the 2015 IEEE Conference on Computer Communications Workshops (INFOCOM WKSHPS).

[6] Sathiyanarayanan M., Rajan S. MYO Armband for physiotherapy healthcare: A case study using gesture recognition application. Proceedings of the 2016 8th International Conference on Communication Systems and Networks (COMSNETS).

[7] Jiang S., Lv B., Guo W., Zhang C., Wang H., Sheng X., Shull P.B. (2018). Feasibility of wrist-worn, real-time hand, and surface gesture recognition via sEMG and IMU Sensing. IEEE Trans. Ind. Inform..

[8] Huang J., Lin S., Wang N., Dai G., Xie Y., Zhou J. (2020). TSE-CNN: A Two-Stage End-to-End CNN for Human Activity Recognition. IEEE J. Biomed. Health Inform..

[9] Kim M., Cho J., Lee S., Jung Y. (2019). Imu sensor-based hand gesture recognition for human-machine interfaces. Sensors.

[10] Kundu A.S., Mazumder O., Lenka P.K., Bhaumik S. (2018). Hand Gesture Recognition Based Omnidirectional Wheelchair Control Using IMU and EMG Sensors. J. Intell. Robot. Syst. Theory Appl..

[11] Wu J., Li X., Liu W., Wang Z.J. (2019). SEMG Signal Processing Methods: A Review. J. Phys. Conf. Ser..

[12] Toledo-Pérez D.C., Martínez-Prado M.A., Gómez-Loenzo R.A., Paredes-García W.J., Rodríguez-Reséndiz J. (2019). A study of movement classification of the lower limb based on up to 4-EMG channels. Electronics.

[13] Qi J., Jiang G., Li G., Sun Y., Tao B. (2019). Intelligent Human-Computer Interaction Based on Surface EMG Gesture Recognition. IEEE Access.

[14] Ma S., Lv B., Lin C., Sheng X., Zhu X. (2021). EMG Signal Filtering Based on Variational Mode Decomposition and Sub-Band Thresholding. IEEE J. Biomed. Health Inform..

[15] Raurale S.A., McAllister J., Del Rincon J.M. (2020). Real-Time Embedded EMG Signal Analysis for Wrist-Hand Pose Identification. IEEE Trans. Signal Process..

[16] Zhang Z., Tang Y., Zhao S., Zhang X. Real-time surface EMG pattern recognition for hand gestures based on support vector machine. Proceedings of the 2019 IEEE International Conference on Robotics and Biomimetics (ROBIO).

[17] Zhang Z., Yu X., Qian J. (2020). Classification of finger movements for prosthesis control with surface electromyography. Sens. Mater..

[18] Ortiz-Echeverri C.J., Salazar-Colores S., Rodríguez-Reséndiz J., Gómez-Loenzo R.A. (2019). A new approach for motor imagery classification based on sorted blind source separation, continuous wavelet transform, and convolutional neural network. Sensors.

[19] Dragomiretskiy K., Zosso D. (2014). Variational mode decomposition. IEEE Trans. Signal Process..

[20] Sanchez-Reyes L.M., Rodriguez-Resendiz J., Avecilla-Ramirez G.N., Garcia-Gomar M.L., Robles-Ocampo J.B. (2021). Impact of EEG Parameters Detecting Dementia Diseases: A Systematic Review. IEEE Access.

[21] Toledo-Pérez D.C., Rodríguez-Reséndiz J., Gómez-Loenzo R.A., Jauregui-Correa J.C. (2019). Support Vector Machine-based EMG signal classification techniques: A review. Appl. Sci..

[22] Geng W., Du Y., Jin W., Wei W., Hu Y., Li J. (2016). Gesture recognition by instantaneous surface EMG images. Sci. Rep..

[23] Smruthy A., Suchetha M. (2017). Real-Time Classification of Healthy and Apnea Subjects Using ECG Signals with Variational Mode Decomposition. IEEE Sens. J..

[24] Ur Rehman N., Park C., Huang N.E., Mandic D.P. (2013). EMD via MEMD: Multivariate Noise-Aided Computation of Standard EMD. Adv. Adapt. Data Anal..

[25] Rehman N.U., Aftab H. (2019). Multivariate Variational Mode Decomposition. IEEE Trans. Signal Process..

[26] Dragomiretskiy K., Zosso D. (2015). Two-dimensional variational mode decomposition. Lect. Notes Comput. Sci. (Incl. Subser. Lect. Notes Artif. Intell. Lect. Notes Bioinform.).

[27] Illankoon P., Tretten P., Kumar U. (2019). Modelling human cognition of abnormal machine behaviour. Hum.-Intell. Syst. Integr..

[28] Chandra N., Vaidya H., Ghosh J.K. (2020). Human cognition based framework for detecting roads from remote sensing images. Geocarto Int..

[29] Shanmuganathan V., Yesudhas H.R., Khan M.S., Khari M., Gandomi A.H. (2020). R-CNN and wavelet feature extraction for hand gesture recognition with EMG signals. Neural Comput. Appl..

[30] Asif A.R., Waris A., Gilani S.O., Jamil M., Ashraf H., Shafique M., Niazi I.K. (2020). Performance evaluation of convolutional neural network for hand gesture recognition using EMG. Sensors.

[31] Shaker A.M., Tantawi M., Shedeed H.A., Tolba M.F. (2020). Generalization of Convolutional Neural Networks for ECG Classification Using Generative Adversarial Networks. IEEE Access.

[32] Luo J., Liu C., Yang C. (2019). Estimation of EMG-Based force using a neural-network-based approach. IEEE Access.

[33] Atzori M., Gijsberts A., Castellini C., Caputo B., Hager A.G.M., Elsig S., Giatsidis G., Bassetto F., Müller H. (2014). Electromyography data for non-invasive naturally-controlled robotic hand prostheses. Sci. Data.

[34] Ning Y., Zhu X., Zhu S., Zhang Y. (2015). Surface EMG decomposition based on K-means clustering and convolution kernel compensation. IEEE J. Biomed. Health Inform..

[35] Lobov S., Krilova N., Kastalskiy I., Kazantsev V., Makarov V.A. (2018). Latent factors limiting the performance of sEMG-interfaces. Sensors.

[36] Tsinganos P., Cornelis B., Cornelis J., Jansen B., Skodras A. Improved Gesture Recognition Based on sEMG Signals and TCN. Proceedings of the ICASSP 2019—2019 IEEE International Conference on Acoustics, Speech and Signal Processing (ICASSP).

[37] Bai L., Zhao Y., Huang X. (2018). A CNN Accelerator on FPGA Using Depthwise Separable Convolution. IEEE Trans. Circuits Syst. II Express Briefs.

[38] Atzori M., Gijsberts A., Kuzborskij I., Elsig S., Hager A.G.M., Deriaz O., Castellini C., Müller H., Caputo B. (2015). Characterization of a benchmark database for myoelectric movement classification. IEEE Trans. Neural Syst. Rehabil. Eng..

[39] Du Y., Jin W., Wei W., Hu Y., Geng W. (2017). Surface EMG-based inter-session gesture recognition enhanced by deep domain adaptation. Sensors.

